# Visual movement impairs duration discrimination at short intervals

**DOI:** 10.1177/17470218231156542

**Published:** 2023-02-21

**Authors:** Nathércia L Torres, São Luís Castro, Susana Silva

**Affiliations:** Center for Psychology at the University of Porto (CPUP), Faculty of Psychology and Educational Sciences, University of Porto, Porto, Portugal

**Keywords:** Duration perception, visual movement, sensory modality, interval length

## Abstract

The classic advantage of audition over vision in time processing has been recently challenged by studies using continuously moving visual stimuli such as bouncing balls. Bouncing balls drive beat-based synchronisation better than static visual stimuli (flashes) and as efficiently as auditory ones (beeps). It is yet unknown how bouncing balls modulate performance in duration perception. Our previous study addressing this was inconclusive: there were no differences among bouncing balls, flashes, and beeps, but this could have been due to the fact that intervals were too long to allow sensitivity to modality (visual vs auditory). In this study, we conducted a first experiment to determine whether shorter intervals elicit cross-stimulus differences. We found that short (mean 157 ms) but not medium (326 ms) intervals made duration perception worse for bouncing balls compared with flashes and beeps. In a second experiment, we investigated whether the lower efficiency of bouncing balls was due to experimental confounds, lack of realism, or movement. We ruled out the experimental confounds and found support for the hypothesis that visual movement—be it continuous or discontinuous—impairs duration perception at short interval lengths. Therefore, unlike beat-based synchronisation, duration perception does not benefit from continuous visual movement, which may even have a detrimental effect at short intervals.

A generally accepted finding in the field of time processing is that time discrimination and sensorimotor synchronisation in humans are more accurate for auditory than for visual stimuli (Grondin & McAuley, 2009; Merchant, Zarco, et al., 2008; Merchant, Zarco, & Prado, 2008; van Wassenhove, 2009). However, recent findings have shown that the auditory advantage is not always present, as previously thought: beat-based synchronisation with *visual stimuli in continuous motion* (bouncing balls) can be as effective as synchronisation with an auditory metronome, while synchronisation with *static visual stimuli* (flashing balls) cannot (Hove, Iversen, et al., 2013; Silva & Castro, 2016; Torres et al., 2019). In this context, Hove, Fairhurst and colleagues (2013) put forward the idea of interaction between modality (auditory vs visual) and continuity (moving vs static stimuli) and showed that moving visual stimuli (bouncing balls, continuous presence) are more efficient than static ones (flashes, discontinuous) in driving synchronisation, whereas in the auditory domain the opposite occurs — discontinuous sounds such as beeps elicit better performance than continuous ones, such as sirens.

The equivalence between beeps and bouncing balls has not yet been demonstrated in timing domains other than beat-based synchronisation, namely in beat-based pure perception. Several studies demonstrated that bouncing balls match beeps in beat-based synchronisation but not in beat-based purely perceptual tasks (Gu et al., 2020; Silva & Castro, 2016; Torres et al., 2019). In Silva and Castro (2016), both flashes and bouncing balls elicited poorer beat perception than beeps at 300 ms base lengths. In Gu et al. (2020), bouncing balls elicited better beat perception than flashes at 700 ms lengths, but they were less efficient than beeps.

Duration perception is a timing domain that dissociates in many ways from beat-based perception. Unlike beat-based, duration perception is absolute and does not rely on an isochronous beat for reference, thus not allowing prediction (Grahn & McAuley, 2009; Grube et al., 2010; McAuley & Jones, 2003; Teki et al., 2011). Available findings regarding the status of bouncing balls in duration perception remain inconclusive. In a previous experiment targeting duration perception with average base lengths of 391 ms (Torres et al., 2019), the authors of this article found no differences between bouncing balls and flashes, but—different from beat-based perception measured with similar intervals (300 ms)—they did not see any differences between flashes and beeps either. The results from Torres et al. (2019) are inconclusive because they were unable to rule out that the equivalent duration discrimination performance across all three stimuli—flashes, balls, and beeps—was due to the base length of intervals, which may have triggered modality-insensitive, high-level processing. According to the *distinct timing hypothesis*, duration discrimination of shorter intervals is sensitive to modality, while discrimination of longer ones is not (Rammsayer et al., 2015; Rammsayer & Troche, 2014; Wiener et al., 2011). The distinct timing hypothesis posits two qualitatively distinct timing networks for temporal processing in shorter versus longer intervals (Rammsayer & Ulrich, 2005). Within this framework, shorter intervals can be considered as sensory-automatic, while longer intervals require higher-level cognitive resources (Rammsayer et al., 2015; Rammsayer & Ulrich, 2011). The distinct timing hypothesis is one of two different answers to a central question in time perception—whether temporal processing at different time ranges relies on common or distinct circuits (Rammsayer et al., 2015; Rammsayer & Troche, 2014; Wiener et al., 2011). The alternative and traditional theory—the *common timing* approach—assumes that temporal perception across all time ranges is based on a single internal clock (Gibbon, 1977, 1992; Grondin, 2010; Lewis & Miall, 2009). Considering the distinct timing hypothesis and the associated empirical studies, it is not impossible that the intervals in Torres et al. (2019) were approached as long ones, and this precluded cross-stimulus differences from emerging. Among the available studies, the boundary between short and long varies. Some refer to sub-second (1,000 ms included) versus supra-second intervals as synonyms for short versus long. This was the case with Ortega and colleagues (2014), who demonstrated that audition dominates vision in bimodal duration perception intervals ranging between 200 and 1,000 ms, even when the visual component is highlighted. In a similar vein, Murai and Yotsumoto (2016) concluded that durations in the subsecond range (400–600 ms) are controlled by both modality-dependent and modality-independent mechanisms, whereas durations in the suprasecond range (2,000–3,000 ms) are mediated by an amodal timing mechanism. In a slightly different approach, other studies classify 1,000 ms as a long interval: Rammsayer and Pichelmann (2018) found that modality effects in duration discrimination were more pronounced for 50 ms (short) intervals than for 1,000 ms (long) intervals. In contrast to the sub-second versus supra-second approach, other studies placed the boundary between short and long at a lower point: Rammsayer (2014) showed large auditory-visual differences in temporal discrimination for intervals shorter than 600 ms, with increased sensitivity for audition compared with vision. When using base durations longer than 600 ms, the auditory-visual difference levelled off, even though a slight superiority of the auditory domain persisted. If it is true that 600 ms sets the boundary between short and long, then at least some of the intervals tested in Torres et al. (2019: *M* = 391, *SD* = 74, range = 167–733 ms) may have been approached as long intervals, meaning that they were insensitive to modality effects. Insensitivity to modality may have impeded differences between auditory (beeps) and static visual (flashes) and moving visual stimuli (bouncing balls) to emerge, thus not resolving the question concerning the status of moving visual stimuli in duration perception.

The present study was designed to clarify the status of bouncing balls in duration perception. We conducted a first experiment (Experiment 1) to test whether differences between bouncing balls, flashes, and beeps require intervals shorter than those used in Torres et al. (2019) to emerge—something that may have impeded a valid analysis of the status of bouncing balls. To that end, we compared beeps, flashes, and bouncing balls using two base lengths: one similar to that of Torres et al. (2019), which we named *medium* (*M* = 326, *SD* = 144, range = 133–733 ms), and a shorter base length (*M* = 157, *SD* = 68, range = 67–333 ms), which we named *short*. If the explanation for the null results of Torres et al. (2019) was that medium stimuli were too long to be sensitive to modality—as predicted by the distinct timing hypothesis—we should see modality effects in duration perception for short, but not for medium intervals. If duration-based perception is equivalent to beat-based synchronisation, flashes should elicit poorer performance than beeps, which would match bouncing balls. If duration-based perception is equivalent to beat-based perception, both flashes and bouncing balls should elicit poorer performance than beeps. Results (see below) showed evidence that length does modulate modality effects (equivalence for bouncing balls, flashes and beeps at medium base lengths), but none of the predicted patterns emerged for short intervals: here, bouncing balls (visual) elicited poorer performance than both flashes (visual) and beeps (auditory). To investigate the potential causes of poorer performances for bouncing balls at short base lengths, we ran a second experiment (Experiment 2). Here, we tested whether a deleterious effect of movement explained the results or, on the contrary, these were better accounted for by lack of realism in the stimulus or to experimental confounds.

## Experiment 1: cross-stimulus differences at short versus medium lengths

### Method

#### Participants

Fifty-one adult volunteers (six men) took part in the experiment (*Mean age* *=* 21.5 years, *SD* = 3.7). All participants were naїve to the purpose of the study, had normal or corrected-to-normal vision, and did not report neurological, motor, or hearing disorders. The study was approved by the local ethics committee (2021/02-01b) and all participants signed informed consent according to the Declaration of Helsinki.

#### Stimuli

**Figure 1.**
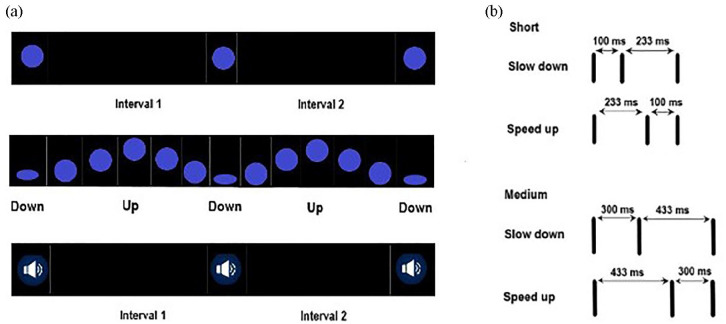
(a) Sequences of flashes (above), bouncing balls (middle) and beeps (below) used in Experiment 1. (b) For each type, half the stimuli were slowing down and the other half speeding up. Stimuli were presented twice—at short versus medium base lengths.

Each stimulus sequence included two time intervals where the second interval could be longer (slow-down sequence) or shorter than the first one (speed-up sequence). The two intervals were defined by three events, which differed according to stimulus type (Figure 1): for auditory sequences, we had three short sinusoidal tones (F0 = 450 Hz, 67 ms, +70 dB) that we refer to as *beeps*. Each three-beep sequence corresponded to a 16 bit, mono audio file at 44.1 kHz sampling frequency. Static visual sequences included three short appearances (*flashes*) of a static blue ball (5% of screen width, 9% of screen height), length of appearance = 67 ms) centred on a black background. The background remained black between flashes. Moving visual sequences used the same blue ball, but now travelling up (to screen centre) and down within a vertical line (*bouncing ball*) on the black screen and squashing at the lower point of the trajectory (an imaginary ground) three times. The ball remained continuously visible on the screen and had a linear trajectory. Visual sequences (flashes and bouncing balls) corresponded to videos at 30 frames per second. Auditory sequences started with 200 ms of silence, and visual flashes with 200 ms of black background. Visual bouncing balls started with a 600 ms interval featuring the falling ball, which squashed at the imaginary ground and marked the onset of the first interval. Visual sequences were displayed in a 46 cm-wide monitor, set to a resolution of 1280 × 1024 pixels, with a refresh rate of 60 Hz.

Temporal sequences made up with beeps, flashes and balls were presented at two different base lengths, which we named *medium* and *short*. For each of the six stimulus type × base length combinations (three stimulus types × two duration levels), we had 14 sequences, half speeding up and half slowing down within each set (7 + 7, Figure 1 and the Online Supplementary Material A and B). *Medium intervals* were similar in length to those of Torres et al. (2019), with an average length of 326 ms and range of 133–733 ms. The average difference between the two intervals in a sequence [average of (interval 2 − interval 1)] was 176 ms (see the Online Supplementary Material A and B). Short intervals ranged between 67 and 333 ms and had an average length of 157 ms. The average difference between intervals was made shorter compared with medium ones (90 ms), in a way that the ratio between average interval difference (see above) and average interval length [average of (interval 1 + interval 2)/2] was kept similar across the two base lengths (~55%). A paired-sample *t*-test on the ratio between average interval difference and average interval length for short versus medium intervals showed no evidence of significant differences, *t*(13) = 0.49, *p* = .63. The travelling distance of the bouncing ball was the same across base durations (10% of screen height).

#### Procedure

We ran the experiment on E-prime 2 (https://pstnet.com/products/e-prime/). Participants sat 55 cm away from a Samsung Syncmaster 957DF monitor, positioned at the eye level. They were asked to judge whether each of 14 sequences for every base duration (medium and short) × stimulus type (balls, beeps, flashes) combination speeded up or slowed down. They responded by pressing keys “1” or “2” on the computer keyboard. Before the experimental phase, there was a training phase in which we showed participants one example of each stimulus type (balls, beeps, and flashes) speeding up and slowing down in every base duration, and then clarified possible doubts about the task. Participants were instructed to focus on the two intervals between the three relevant events—the appearance of flashes, the onset of beeps and, for balls, the moment when balls squashed on the imaginary ground. We told them that, if the second interval was longer than the first, they should respond “slow down,” otherwise the response would be “speed up.”

The six combinations between the three-stimulus types (balls, beeps and flashes) and the two base durations (medium and short) were ordered in six different ways, based on stimulus type and length (see the Online Supplementary Material C). For each of these orders, we created two conditions: one in which the left key (“1”) meant speed up and another where it meant slow down. Each participant was assigned to one of these 12 conditions (6 orders × 2 keys). Participants wore headphones in all tasks—in the auditory tasks to listen to the stimuli, and in the visual ones to minimise any noise from outside the room. A central fixation point was presented before every stimulus onset. For auditory stimuli, the fixation point remained visible throughout the trials to prevent attentional shifts. In total, participants responded to 84 trials (14 trials × 6 conditions).

For exploratory purposes, we also administered the Portuguese version of Goldsmiths Musical Sophistication Index (Gold-MSI, Lima et al., 2020), a self-report measure of how individuals engage with music that includes, but is not limited to, musical training. The Gold-MSI self-report questionnaire has five sub-scales for different facets of musical sophistication: musical training, active engagement with music, perceptual abilities, singing abilities, and emotions, all viewed as components of musical sophistication. A general musical sophistication factor that incorporates aspects from the five sub-scales is also included. Studies on the relation between duration perception (non-musical time) and musical skills are not abundant, in contrast to studies that focused on beat-based perception (musical time) and showed a positive effect of musical training (e.g., Chen et al., 2008). One study on duration perception demonstrated that musical expertise improves auditory but not tactile duration discrimination (Güçlü et al., 2011), thus pointing to a modality-specific advantage in musicians. However, to our knowledge, comparisons across auditory, moving visual, and static visual stimuli have not yet been carried out. By correlating Gold-MSI scores with duration discrimination performance across our three stimulus types, we expected to shed more light on this matter. The length of the experiment including questionnaires ranged between 35 and 45 min.

#### Statistical analysis

The analysis was run with JASP (Version 0.14; JASP Team, 2020). Performance in the duration-based perception task was approached with *d*′ measures (Stanislaw & Todorov, 1999). The hit ratio comprises the proportion of speed up trials to which subject responded speed up and the false alarm ratio comprises the proportion of slow down trials to which subjects responded speed up. First, we compared *d*′ values per condition against zero, using one-sample *t*-tests or Wilcoxon tests depending on the normality of the data as tested with Shapiro-Wilk tests (Wilcoxon used for non-normal distributions and *t*-test otherwise). The main analysis was a 3 × 2 within-subjects analysis of variance with stimulus type (beeps vs flashes vs bouncing balls) and base duration (short vs medium) as factors. For all post hoc comparisons, Bonferroni-corrected *t*-tests were applied. Again, when violations of normality were present, we cross-checked the results with non-parametric tests (Friedman and Wilcoxon tests). According to sensitivity power analyses carried out with G*Power (Faul et al., 2007), the minimum effect sizes for repeated measures tests (design 2 × 3: length × stimuli) that our tests were able to detect reliably with 80% power (alpha = .05) were in the small range (η^2^p < .05; η^2^p = .02 in our case).

Possible associations between musical sophistication and temporal performance were approached employing correlation analyses and Bonferroni correction for multiple tests. According to sensitivity power analyses, the minimum detectable effect size for the correlations (80% power, alpha = .05) was in the small range (<.30; *r* = .29).

The data for experiment 1 are available at https://osf.io/dzphx/files.

### Results

The *d*’ values differed significantly from zero in all conditions (all *p*s < .001), showing that discrimination between speed up and slow down sequences was above chance levels for all stimulus types and base duration levels (see Table 1).

**Table 1.** One-sample *t*-tests against zero for the six stimulus type × length conditions.

Stimulus	Test	Statistic	Degrees of freedom	*p*	Effect size	Mean *d*′	*SD*
Beep short	Student	9.04	50	<.001	1.26	1.04	0.82
Beep medium	Wilcoxon	1,258.00	50	<.001	0.97	1.39	0.82
Ball short	Wilcoxon	916.00	50	<.001	0.77	0.21	0.32
Ball medium	Wilcoxon	1,072.00	50	<.001	0.98	1.58	1.03
Flash short	Student	8.22	50	<.001	1.15	0.86	0.75
Flash medium	Wilcoxon	1,275.00	50	<.001	1.00	1.73	0.69

*SD*: standard deviation.

For the Student *t*-test, effect size is given by Cohen’s *d*. For the Wilcoxon test, effect size is given by the matched rank biserial correlation.

**Figure 2.**
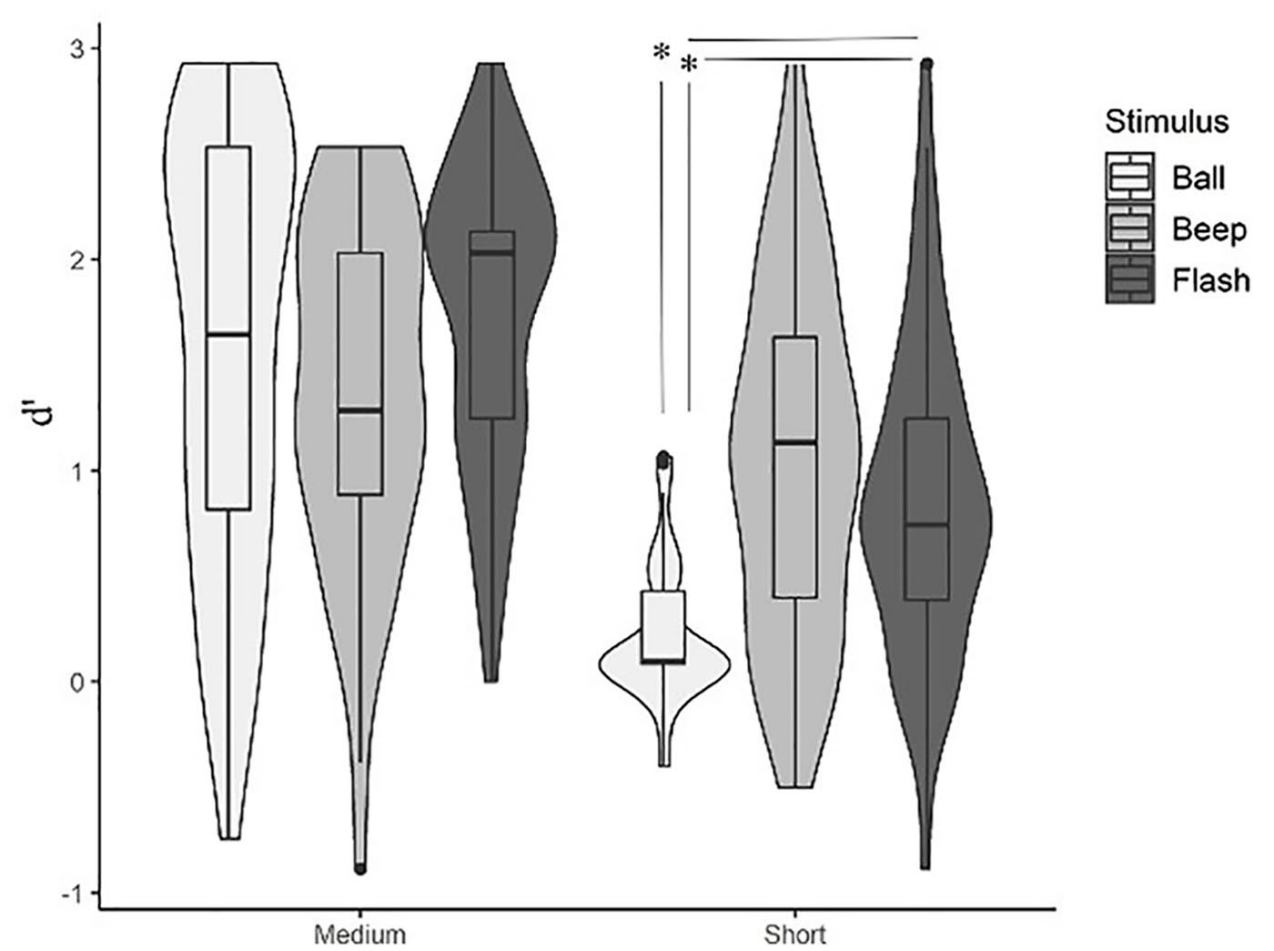
Violin plots representing the discrimination between speed-up and slow-down sequences as a function of stimulus type (auditory: beep; moving visual: ball; static visual: flash) and base duration (short: 67–333 ms; medium: 167–733 ms). Boxplots are aggregated by base duration.

Concerning the repeated-measures analysis of variance (ANOVA) results (Figure 2), the main effect of stimulus type was significant, *F*(1.80, 90.17) = 10.09, *p* < .001, η^2^p = .16. Post hoc Bonferroni-corrected *t*-tests indicated better performance in flashes and beeps compared with balls (flashes vs balls: *p* < .001, *d* = 0.59; beeps vs balls: *p* = .003, *d* = 0.47). No significant difference could be established between beeps and flashes, *p* > .999, *d* = 0.11. A non-parametric Friedman test of differences among repeated measures was also conducted and showed a significant effect of stimulus type, χ^2^ (2) = 7.171; *p* = .02, Kendall’s *W* = .045. A significant main effect of base duration was also found, *F*(1, 50) = 187.26, *p* < .001, η^2^p = .78, indicating better performance in medium than in short base durations, *p* < .001, *d* = 1.9. The non-parametric Friedman test rendered a significant effect of base duration, χ^2^ (1) = 41.491; *p* < .001, Kendall’s *W* = .327.

Critical to our goal, the interaction between stimulus type and base duration on *d*′ values was significant, *F*(2, 50) = 12.782, *p* < .001, η^2^p = .20, indicating that discrimination between stimulus types was modulated by base length (Table 2). For medium base lengths, there were no statistically significant differences between stimulus types, also confirmed by the Wilcoxon signed rank test. For short base lengths, balls elicited poorer performance than both beeps and flashes, and beeps and flashes did not differ from one another. The non-parametric Wilcoxon test also indicated that subject’s performance was poorer in balls when compared with beeps and flashes.

**Table 2.** Parametric and non-parametric post hoc pairwise comparisons across stimulus types according to length.

Length	Comparison	*t*(50)	*p*	Corrected *p*	Cohen’s *d*	
Medium	Ball = Flash	1.077	.287	>.999	0.15	
	Ball = Beep	1.135	.262	>.999	0.16	
	Flash = Beep^a^	2.358	.022	.132	0.33	
Medium	Non-parametric comparison	*z*	*p*	Corrected *p*	Effect size	Wilcoxon
	Ball = Flash	0.776	.441	>.999	0.13	469.500
	Ball = Beep	1.328	.186	>.999	0.21	746.000
	Flash = Beep^a^	2.092	.37	>.999	0.35	732.000
Length	Comparison	*t*(50)	*p*	Corrected *p*	Cohen’s *d*	
Short	**Ball** **<** **Flash**	5.489	<.001	<.001	0.79	
	**Ball** **<** **Beep**	6.617	<.001	<.001	0.93	
	Flash = Beep	1.420	.162	.972	0.19	
Short	Non-parametric comparison	*z*	*p*	Corrected *p*	Effect size	Wilcoxon
	**Ball** **<** **Flash**	4.574	<.001	<.001	0.75	1,034.000
	**Ball** **<** **Beep**	5.010	<.001	<.001	0.81	1,158.500
	Flash = Beep	1.593	.112	>.999	0.26	472.500

Bold text indicates significant differences.

a Flashes showed increased discrimination compared with beeps, but the difference was non-significant after Bonferroni corrections.

Associations between musical sophistication and duration discrimination performance are summarised in Table 3. After Bonferroni corrections, two marginally significant correlations were found: a positive correlation between medium beeps and the perceptual abilities subscale of Gold-MSI (*r* = .344, *p* = .078), and a negative one between short balls and the emotions subscale (*r* = .349, *p* = .072). Both became marginal after corrections for multiple correlations were applied (6 correlations, one per scale, Table 3).

**Table 3.** Correlations between Gold-MSI subscales and performance on duration discrimination.

Stimulus	Interval duration	General sophistication	Active engagement	Perceptual abilities	Singing abilities	Emotions	Musical training
Beep	Short	0.03	0.11	0.10	0.01	0.19	0.20
	Medium	0.04	0.02	0.34*	0.02	0.19	0.10
Ball	Short	0.22	0.23	0.22	0.07	0.35*	0.16
	Medium	0.008	0.08	0.03	0.10	0.10	0.03
Flash	Short	0.12	0.05	0.11	0.02	0.09	0.16
	Medium	0.09	0.15	0.04	0.02	0.04	0.04

Gold-MSI: Goldsmiths Musical Sophistication Index.

Bold values indicate marginal correlations.

* *p* < .10 after Bonferroni correction.

### Discussion

The goal of Experiment 1 was to test whether differences between bouncing balls, flashes, and beeps in duration perception emerge in short but not medium intervals. A previous study of ours (Torres et al., 2019) showed no differences between the three stimulus types in duration perception tasks, but this could have been due to the amodal timing mechanism elicited by our longer intervals, as predicted by the distinct timing hypothesis for duration perception. Therefore, in the present experiment, we tested intervals similar to those of Torres et al. (2019), which we named medium, contrasting these with shorter intervals. We expected modality differences (better performance for audition than vision) to emerge only in short intervals and, from this, be able to clarify the status of moving visual stimuli in temporal perception.

Our results supported the prediction that modality differences would be absent in medium intervals, thus replicating our earlier findings (Torres et al., 2019). As for short intervals, differences emerged, though not totally consistent with the modality-related predictions of the distinct timing hypothesis: though short intervals elicited better performance in auditory (beeps) than moving visual stimuli (bouncing balls), auditory and static visual stimuli (flashes) were equivalent. Considering that extant evidence in favour of the distinct timing hypothesis has been substantiated in the advantage of auditory over *static* visual stimuli (Barne et al., 2018; Rammsayer, 2014; Rammsayer et al., 2015), we cannot say that we supported the hypothesis as it was framed in the past, that is, based on effects of modality per se.

Having established that participants had poorer performances in bouncing balls than in flashes and beeps when the circumstances (short lengths) allow differences to emerge, the next question is why this occurs. One explanation concerns a potential experimental confound and (1) may relate to the fact that our short balls were not corrected for travelling distance (they travelled to the same height as medium balls). This may have made the task more difficult due to the increased travelling speed.

Apart from experimental confounds, two other explanations may be considered. A second possibility is that (2) short bouncing balls were taxed by the non-natural, non-ecological characteristics of fast tempo, similar to what happens in beat-based synchronisation (Gan et al., 2015). Bouncing balls are real-life objects subjected to mechanical constraints, while flashes are not. Unlike a fast flash, which is as (un)natural as a slower one, balls are not expected to bounce as fast as we made them to. The other explanation is that (3) movement per se was the critical element. It is known that stimulus movement (change in spatial location) may distort duration perception for visual stimuli (Brown, 1995; Kanai et al., 2006; Kaneko & Murakami, 2009). One reason for this to happen is that, when confronted with moving stimuli, participants must perform additional operations such as monitoring different positions or shifting attention between two locations (Cicchini & Morrone, 2009). If these additional computations are made online, as time intervals unfold, they may be particularly overloading when intervals are short. Thus, they are likely to add noise in duration judgements under these circumstances. It has also been suggested that high velocities per se, that is, regardless of attention shifts, maximise time distortions (Gorea & Kim, 2015; Kaneko & Murakami, 2009). Therefore, it is also possible that the increased velocities of our moving stimuli (associated with short intervals) had a direct impact on the poor results we saw for short bouncing balls and short flashes.

To investigate these three possibilities, we conducted a second experiment (Experiment 2). To (1) rule out experimental confounds related to travelling distance, we compared medium balls, short unadjusted balls and a newly created set of sequences—short balls adjusted for travelling distance. If unadjusted travelling distances were responsible for degraded performance with bouncing balls at short lengths, we should see equivalent performances for medium and short adjusted balls as well as increased performance for adjusted balls, compared with non-adjusted. To decide between explanations based on the (2) non-realistic features of fast bouncing balls and those based on the (3) more general detrimental effect of visual movement at short base lengths, we created a new stimulus type—moving flashes, which alternated between up and down positions on screen. Fast-moving flashes are not expected to be less natural than fast static flashes. Therefore, if both moving flashes and balls elicited lower performances than static flashes at short base lengths, there would be no reason to explain the disadvantage of short bouncing balls based on non-realistic features, and this would be consistent with the possibility that movement per se impairs duration perception at short base lengths. On the contrary, if discrimination in balls but not in moving flashes was poorer than in static flashes at short intervals, we would not be able to rule out the role of non-realistic features.

## Experiment 2: why bouncing balls elicited lower performance at short intervals

### Method

#### Participants

Fifty-four adult volunteers (18 men) took part in the experiment (*Mean age* *=* 28.7 years, *SD* = 10). All participants were naїve to the purpose of this study, had normal or corrected-to-normal vision, and did not report neurological, motor, or hearing disorders. The study was approved by the local ethics committee (2021/02-01b) and the participants signed informed consent according to the Declaration of Helsinki.

#### Stimuli

**Figure 3.**
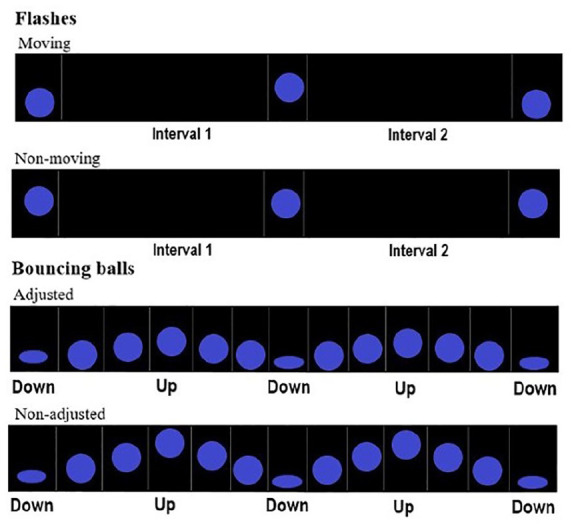
Sequences of moving flashes (above) and short balls (below) adjusted for travelling distance used in Experiment 2. For comparison purposes, the non-transformed stimuli used in Experiment 1 are presented below.

We created two new stimulus types, derived from short bouncing balls and flashes: short bouncing balls with adjusted (shorter) travelling distance, and moving flashes (with both short and long base intervals, Figure 3). In short bouncing balls with adjusted travelling distance (*short adjusted balls*, hereafter), the highest point of the trajectory was lowered, such that the ball travelled along 5% of screen height (against 10% of screen height in short non-adjusted and medium balls). In *moving flashes*, the position of the static ball alternated between the unadjusted (central) position and a lower position, corresponding to the lowest (squashing) point of the bouncing ball.

Short (non-adjusted) bouncing balls, as well as medium and short flashes, had the characteristics described in Experiment 1.

#### Procedure

We ran the experiment online on Psychopy^3^/Pavlovia (www.psychopy.org). In the dissemination phase of the study, participants were informed that the experiment only worked on a computer, and it was not possible to run it on a cell phone or tablet. Before beginning the study, they consented to participate by pressing the backspace key after reading an informed consent web page. Thereafter, participants were given an example of each stimulus speeding up and slowing down and how to respond to it, as in the following sentence: “Now you will see an example of a moving flash speeding up. The correct answer is “r.” Press the key r to go to the next example” (similarly, mutatis mutandis, for the flash slowing down, where the key to press was “l”). Given that the experiment was run online, and we had little control over participants’ attention to using the right response key, we chose an intuitive key for each response type and used it for all participants. Specifically, for slow-down responses, they had to press “l” (from *lento*, Portuguese for slow) and “r” for speed-up (from *rápido*, Portuguese for speedy).

The seven sequence types (medium balls, short balls, short adjusted balls, medium flashes, short flashes, medium moving flashes, and short moving flashes) were organised in four different orders (see the Online Supplementary Material D). These orders were balanced across participants. In total, participants responded to 98 trials (14 trials × 7 conditions).

#### Statistical analysis

As in Experiment 1, the analysis was run with JASP (Version 0.14; Jasp Team, 2020) with repeated measures ANOVAs on *d*′ measures.

After testing discrimination values against zero for all the seven conditions (medium, short unadjusted, and short adjusted balls; medium and short flashes; medium and short moving flashes), the first level of analysis addressed the effects of adjusting the bouncing ball travelling distance in short balls: would this decrease the disadvantage of short balls compared with medium ones? To determine this, we compared medium, short and short adjusted balls with a repeated-measures ANOVA (one factor with three levels). According to sensitivity power analyses carried out with G*Power (Faul et al., 2007), the minimum effects sizes (3 levels of stimuli) that our tests were able to detect reliably with 80% power (alpha = .05) were in the small range (η^2^p < .05; η^2^p = .02 in our case).

The second level addressed the interaction between movement (present in balls and moving flashes, absent in flashes) and length. To that end, we performed a 3 × 2 repeated measures ANOVA with stimulus type (balls, flashes, moving flashes) and length (medium, short) as factors. A power analysis for this interaction (3 × 2 design) was performed and the minimum effects sizes that our tests were able to detect reliably with 80% power (alpha = .05) were in the small range (η^2^p < .05: η^2^p = .02 in our case).

As in Experiment 1, we cross-checked the results with non-parametric tests whenever there were violations of normality.

The data for experiment 2 are available at https://osf.io/dzphx/files.

### Results

The *d*′ values differed significantly from zero in all conditions (all *ps* < .05) except short moving flashes, where discrimination was only marginally above chance (Table 4).

**Table 4.** One-sample *t*-tests against zero for the seven conditions of experiment 2.

Stimulus	Test	Statistic	Degrees of freedom	*p*	Effect size^a^
Medium ball	Student	5.15	53	<.001	0.702
Short ball, adjusted	Wilcoxon	541.000	53	0.007	0.460
Short ball, unadjusted	Wilcoxon	265.50	53	0.002	0.634
Medium flash	Wilcoxon	1,143.50	53	<.001	0.867
Medium moving flash	Wilcoxon	936.50	53	<.001	0.810
Short flash	Student	6.18	53	<.001	0.841
Short moving flash	Wilcoxon	632.50	53	.098	0.222

a For the Student *t*-test, effect size is given by Cohen’s *d*. For the Wilcoxon test, effect size is given by the matched rank biserial correlation.

**Figure 4.**
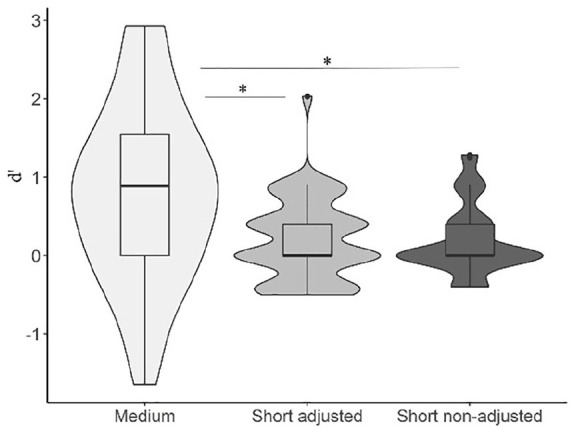
Violin plots representing the discrimination between speed-up and slow-down sequences as a function of base duration (short: 67–333 ms; medium: 167–733 ms) and adjusted travelling distance (short: adjusted vs non-adjusted).

The repeated measures ANOVA with medium, short and short adjusted balls showed a main effect of ball type, *F*(1.38, 71.7) = 14.26, *p* < .001, η^2^p = .21, see Figure 4. A non-parametric Friedman test among balls yielded a significant difference, χ^2^ (2) = 24.720; *p* < .001; Kendall’s *W* = .23. Post hoc Bonferroni-corrected *t*-tests showed no difference between the original short balls and short balls adjusted for travelling distance, *p* > .999. The Conover’s post hoc comparison between short- and short adjusted balls rendered no significant difference, *p* > .999. Medium balls were more efficient than both short balls and short adjusted balls, all *p*s < .001 and *d*s > 0.63.

**Figure 5.**
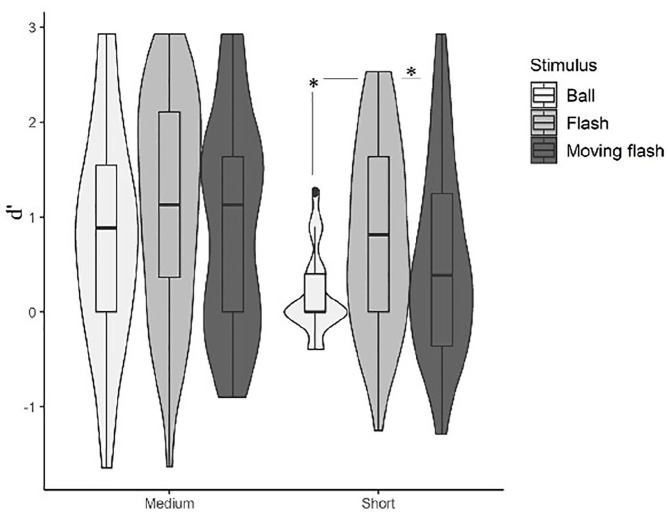
Violin plots representing the discrimination between speed-up and slow-down sequences as function of stimulus type (balls, static flashes, moving flashes) and base length (short and medium). Boxplots are aggregated by base duration.

The three-way repeated measures ANOVA with balls, flash movement (non-moving vs moving, Figure 5) and base length (medium vs short) as factors showed a main effect of stimulus type, *F*(2, 104) = 11.627, *p* < .001, η^2^p = .18, with static flashes eliciting better performance than both moving flashes and balls, *p*_flashxmovingflash_ = .002, *d*_flashxmovingflash_ = 0.48; *p*_flashxball_ < .001, *d*_flashxball_ = 0.63. No significant differences could be established between balls and moving flashes, *p* = .79, *d* = 0.15. There was also a main effect of base length, with increased discrimination for medium intervals, *F*(1, 52) = 21.315, *p* < .001, η^2^p = .29, *p*_mediumxshort_ < .001, *d* = 0.63. The non-parametric Friedman test rendered a significant effect of stimulus types, χ^2^ (2) = 10.137; *p* = .006; Kendall’s *W* = .071, and a significant effect of base length, χ^2^ (1) = 12.659; *p* < .001; Kendall’s *W* = .141.

The interaction between visual stimulus type and base length did not reach significance, *p* = .149. Nevertheless, given that it was near-to-marginal, and that the results of tests against zero pointed to a particular disadvantage of short moving flashes (an index that moving flashes could be particularly affected by length), we moved on with pairwise comparisons (Table 5).

**Table 5.** Parametric and non-parametric post hoc pairwise comparisons across stimulus types according to length.

Length	Comparison	*t*(52)	*p*	Corrected *p*	Cohen’s *d*	
Medium	Ball = Flash	2.553	.014	.084	0.34	
	Ball = Moving flash	0.996	.324	> .999	0.14	
	Moving flash = Flash^a^	1.703	.094	> .999	0.23	
Medium	Non-parametric comparison	*z*	*p*	Corrected *p*	Effect size	Wilcoxon
	Ball = Flash	1.759	.079	.474	0.298	379.500
	Ball = Moving flash	1.163	.247	> .999	0.199	414.500
	Moving flash = Flash	1.443	.151	.906	0.253	592.500
Short	Comparison	*t*(52)	*p*	Corrected *p*	Cohen’s *d*	
	**Ball** **<** **Flash**	4.880	<.001	<.001	0.67	
	Ball = Moving flash	0.611	.544	>.999	0.08	
	**Moving flash** **<** **Flash**	3.619	<.001	<.001	0.49	
Short	Non-parametric comparison	*z*	*p*	Corrected *p*	Effect size	Wilcoxon
	**Ball** **<** **Flash**	4.058	<.001	<.001	0.695	158.000
	Ball = Moving flash	0.158	.879	>.999	0.027	531.500
	**Moving flash** **<** **Flash**	3.463	<.001	<.001	.586	857.500

Bold text indicates significant differences.

a Flashes showed increased discrimination compared with balls, but the difference was non-significant after Bonferroni corrections.

### Discussion

In this second experiment, we wanted to examine potential reasons for the decreased efficiency of short-interval balls compared with short-interval flashes that we saw in Experiment 1. First, we investigated whether this might be due to unadjusted travelling distances (we had kept the same distance for medium and short balls in Experiment 1). Second, we wanted to confront two other explanations, unrelated to experimental confounds: one was that shorter intervals made bouncing balls look unnatural (unlike flashes, which can be both fast or slow in the real world), and the other was that fast bouncing balls represented the difficulty of managing movement at short time intervals.

We ruled out the confound related to travelling distance, since adjusted short balls elicited the same performance as unadjusted ones. Regarding the two other potential explanations—non-realistic movement of fast bouncing balls versus detrimental role of movement per se at short intervals– we found evidence in favour of the second possibility: both balls and moving flashes underperformed non-moving flashes—and these effects were specific to short base durations. Bouncing balls and moving flashes imply movement, but only bouncing balls (a real-life object) are likely to be harmed by lack of realism. Therefore, though we may not rule out other possibilities (something common to balls and moving flashes, other than movement) to account for degraded performance at short intervals, lack of realism was likely not an explanation, and the role of movement per se is consistent with our findings.

## General discussion

Our goal was to better understand the status of visual stimuli with movement (bouncing balls) in duration processing. In Experiment 1, we tested whether short, but not medium intervals allowed differences between beeps, flashes and bouncing balls to emerge, and we found evidence in favour of this: while medium intervals showed no cross-stimulus, differences, participant’s discrimination in bouncing balls was poorer than in flashes and beeps in short intervals. The equivalence between bouncing balls, beeps and static flashes at medium interval durations is consistent with our previous findings (Torres et al., 2019), suggesting that our inconclusive results may have been due to the base interval lengths we used.

In Experiment 2, we tested potential explanations for the disadvantage of bouncing balls at short intervals. First, we ruled out that this resulted from experimental confounds related to the unadjusted travelling distance of short-length balls. Second, we tested between two alternative mechanisms, and investigated whether short-length balls were either affected by the non-natural movement of fast bouncing, or if they were simply affected by movement. To that end, we created another type of moving visual stimulus—the moving flash, which was unlikely to be affected by realism-related issues. We found that both moving flashes and bouncing balls elicited worse performances than static flashes in short (but not medium) intervals, suggesting that the disadvantage of balls at short lengths relates to movement per se, and not to the presence of unnatural movement. The literature includes two different hypotheses on the detrimental effect of movement at short interval lengths: The need for attention shifts (Brown, 1995; Cicchini & Morrone, 2009; Kanai et al., 2006; Kaneko & Murakami, 2009) and the impact of high velocities (Gorea & Kim, 2015; Kaneko & Murakami, 2009). Examining attention shifts (eye movements) and manipulating velocity changes (velocity can be kept constant, if distance is changed) in future studies with bouncing balls and moving flashes could shed some light on this.

The finding that both bouncing balls and moving flashes elicited lower performances than static visual stimuli suggests that participants’ judgements were not hindered by the presence of filled (vs empty) intervals. In filled intervals, the stimulus remains visible throughout the interval’s duration (e.g., a circle that remains on-screen), while in empty ones the stimulus only marks the onset and offset of the interval (what we did with flashes and moving flashes). The literature has shown lower performance for the perception of filled intervals—especially short ones (Grondin, 1993; Grondin et al., 1998). Bouncing balls potentially define filled intervals, but moving flashes do not—they form empty intervals. If both bouncing balls and moving flashes had negative effects on performance, this can mean one of two things: either the opposition between filled and empty intervals was irrelevant in our paradigm, or—most likely—the bouncing ball does not match the characteristics of what has been considered a filled interval because of the embedded movement. Future comparisons between classic filled intervals (static circles remaining on screen) and bouncing balls could further clarify this.

We investigated the status of bouncing balls in duration perception with the ultimate goal of continuing to map its status in time processing, this including beat versus duration processing, as well as perception versus production (synchronisation). Available bouncing ball-related research on beat perception and production has focused on intervals not shorter than those we named medium (not shorter than 300 ms), and thus the comparisons we make here must be limited to our findings on medium intervals. Bouncing balls have proved to be as efficient as auditory beeps in beat-based synchronisation, but less efficient than beeps in beat-based perception (Gu et al., 2020; Silva & Castro, 2016; Torres et al., 2019). In the current study, medium-length bouncing balls were equivalent to beeps, thus apparently resembling beat-based synchronisation. Nevertheless, while beat-based synchronisation has been showing clear disadvantages for flashes, duration perception has not (Torres et al., 2019; current study). In sum, medium-length bouncing balls do not challenge the apparent sensory independence of duration perception for medium/long stimuli (current study), and they seem to benefit from action (higher status in beat synchronisation than in beat perception). This raises one question: could it be that action combined with duration (duration-based synchronisation) also boosts the effectiveness of bouncing balls? Would duration-based synchronisation with medium intervals show any superiority of bouncing balls over flashes? Clarifying this would improve our understanding of the status of moving visual stimuli in time processing. Our findings on short-length bouncing balls cannot be compared with other studies since we could find none in the literature. Further comparisons across stimulus types at short base lengths are needed.

Our main question concerned the status of bouncing balls in duration perception, but, to address it, we also had to address the distinct timing hypothesis—that is, the hypothesis that shorter but not longer intervals elicit modality effects. As we stated above, the idea that longer intervals are insensitive to modality when it comes to perceiving durations was supported by our results, in that performance was equivalent across flashes and beeps. However, its counterpart idea that audition outperforms vision in shorter intervals was not supported: beeps (auditory) and flashes (visual) elicited equivalent outcomes. In this sense, this specific set of findings supports the alternative, common timing hypothesis (e.g., Lewis & Miall, 2009). Rather than modality (audition vs vision), the presence of visual movement seemed to make the difference in short intervals, with visual movement showing a detrimental effect in short intervals. These findings cannot be related to the distinct- versus common-timing hypothesis, in that differences between balls and beeps go beyond modality.

Alongside our main concern with the status of moving visual stimuli in duration perception, our exploratory approach to the relations between musical sophistication and duration perception showed two correlations which, despite being marginal, may feed future research. First, duration perception for beeps correlated positively with musical training, something expected due to known advantages of musicianship concerning auditory-specific duration discrimination (Güçlü et al., 2011). Bouncing balls gave rise to one intriguing correlation: a negative correlation with the subscale of emotions. Could it be that a general propensity to recognise emotions (embedded in this subscale) impacts perception, maybe animacy perception, and complicates the task of detecting short durations conveyed by a moving visual stimulus? This question adds to the many questions raised in the current study, which we hope will get future research attention. Among these, a priority will be the replication of our findings.

## Supplemental Material

sj-docx-1-qjp-10.1177_17470218231156542 – Supplemental material for Visual movement impairs duration discrimination at short intervals Click here for additional data file. Supplemental material, sj-docx-1-qjp-10.1177_17470218231156542 for Visual movement impairs duration discrimination at short intervals by Nathércia L Torres, São Luís Castro and Susana Silva in Quarterly Journal of Experimental Psychology
