# Supplementary material for: Visual movement impairs duration discrimination at short intervals
Source: Q J Exp Psychol (Hove). 2023 Feb 21;77(1):57–69. doi: 10.1177/17470218231156542 (PMC10712207; doi:10.1177/17470218231156542)
Supplement: sj-docx-1-qjp-10.1177_17470218231156542 – Supplemental material for Visual movement impairs duration discrimination at short intervals [file sj-docx-1-qjp-10.1177_17470218231156542.docx]

Supplementary Material for:

Visual Movement Impairs Duration Discrimination at Short Intervals

Nathércia L. Torres, São Luís Castro, and Susana Silva

Center for Psychology at the University of Porto, Faculty of Psychology and Educational Sciences, University of Porto, Porto, Portugal

Nathércia L. Torres <https://orcid.org/0000-0003-0319-0236>

São Luís Castro <https://orcid.org/0000-0002-1487-3596>

Susana Silva <https://orcid.org/0000-0003-2240-1828>

Correspondence concerning this article should be addressed to Nathércia L. Torres, Center for Psychology at the University of Porto (CPUP), Faculty of Psychology and Education Sciences, University of Porto, Rua Alfredo Allen, 4200 - 135 Porto, Portugal. Email: [up201602198@edu.fpce.up.pt](mailto:up201602198@edu.fpce.up.pt)
The data for the experiments reported here are available at <https://osf.io/dzphx/files>.

**Acknowledgments**

This work was supported by the Portuguese Foundation for Science and Technology under grants UIDB/00050/2020, PD/BD/150479/2019 and PTDC/PSI-GER/5845/2020.

**Supplementary Material A: Stimulus sequences for speed up and slow down (ms) in short base duration**

|  | Type | Interval 1 | Interval 2 | Difference |
| --- | --- | --- | --- | --- |
| 1 | Speed up | 333 | 100 | 233 |
| 2 | Speed up | 200 | 67 | 133 |
| 3 | Speed up | 133 | 67 | 66 |
| 4 | Speed up | 233 | 133 | 100 |
| 5 | Speed up | 200 | 133 | 67 |
| 6 | Speed up | 233 | 200 | 33 |
| 7 | Speed up | 100 | 67 | 33 |
| 8 | Slow down | 100 | 233 | -133 |
| 9 | Slow down | 67 | 200 | -133 |
| 10 | Slow down | 67 | 133 | -66 |
| 11 | Slow down | 133 | 233 | -100 |
| 12 | Slow down | 133 | 200 | -67 |
| 13 | Slow down | 200 | 233 | -33 |
| 14 | Slow down | 100 | 167 | -67 |
| Average interval |  |  |  | 157 |
| Average difference |  |  |  | 90.2 |
| Average difference/interval |  |  |  | 0.57 |

**Supplementary Material B: Stimulus sequences for speed up and slow down (ms) in medium base duration**

|  | Type | Interval 1 | Interval 2 | Difference |
| --- | --- | --- | --- | --- |
| 1 | Speed up | 433 | 300 | 133 |
| 2 | Speed up | 300 | 167 | 133 |
| 3 | Speed up | 733 | 167 | 566 |
| 4 | Speed up | 467 | 300 | 167 |
| 5 | Speed up | 433 | 133 | 300 |
| 6 | Speed up | 467 | 300 | 167 |
| 7 | Speed up | 200 | 167 | 33 |
| 8 | Slow down | 300 | 433 | -133 |
| 9 | Slow down | 167 | 300 | -133 |
| 10 | Slow down | 433 | 467 | -34 |
| 11 | Slow down | 300 | 467 | -167 |
| 12 | Slow down | 133 | 433 | -300 |
| 13 | Slow down | 300 | 467 | -167 |
| 14 | Slow down | 167 | 200 | -33 |
| Average interval |  |  |  | 326 |
| Average difference |  |  |  | 176 |
| Average difference/interval |  |  |  | 0.53 |

**Supplementary Material C: Stimulus type and base length combinations in experiment 1**

|  | Stimulus type | Base length |
| --- | --- | --- |
| Version 1 | Ball-Beep-Flash | Medium  Short |
| Version 2 | Beep-Ball-Flash | Medium  Short |
| Version 3 | Flash-Ball-Beep | Medium  Short |
| Version 4 | Ball-Beep-Flash | Short  Medium |
| Version 5 | Beep-Ball-Flash | Short  Medium |
| Version 6 | Flash-Ball-Beep | Short  Medium |

Implemented counterbalancing of stimulus type and base length. The stimulus type presentation follows an order in which visual and audio stimulation came first. For each base length the stimulus presentantion followed the same order.

**Supplementary Material D: Stimulus type and base length combinations in experiment 2**

|  |  | Stimulus type | Base length |  |
| --- | --- | --- | --- | --- |
| Version 1 |  | Ball  Flash | Medium – short – short adjusted  Medium – medium moving – short – short moving |  |
| Version 2 |  | Ball  Flash | Short – short adjusted – medium  Short – short moving – medium – medium moving |  |
| Version 3 |  | Flash  Ball | Medium – medium moving – short – short moving  Medium – short – short adjusted |  |
| Version 4 |  | Flash  Ball | Short – short moving – medium – medium moving  Short – short adjusted - medium |  |

Implemented counterbalancing of stimulus type and base length. The stimulus type presentation follows an order in which balls and flashes stimulations came first.

**Supplementary Material E: Descriptive statistics of experiment 1**

| Stimulus | Mean | *SD* | SE | Minimum | Maximum |
| --- | --- | --- | --- | --- | --- |
| Beep short | 1.04 | 0.82 | 0.11 | -0.53 | 2.93 |
| Beep medium | 1.39 | 0.82 | 0.11 | -0.88 | 2.53 |
| Ball short | 0.21 | 0.32 | 0.04 | -0.40 | 1.06 |
| Ball medium | 1.58 | 1.03 | 0.14 | -0.74 | 2.93 |
| Flash short | 0.86 | 0.75 | 0.10 | -0.88 | 2.93 |
| Flash medium | 1.73 | 0.69 | 0.09 | 0.00 | 2.93 |

**Supplementary Material F: Descriptive statistics of experiment 2**

| Stimulus | Mean | *SD* | SE | Minimum | Maximum |
| --- | --- | --- | --- | --- | --- |
| Ball medium | 0.78 | 1.12 | 0.15 | -1.64 | 2.93 |
| Ball short, adjusted | 0.19 | 0.51 | 0.07 | -0.50 | 2.03 |
| Ball short,  unadjusted | 0.19 | 0.44 | 0.06 | -0.39 | 1.28 |
| Flash medium | 1.14 | 1.12 | 0.15 | -1.63 | 2.93 |
| Flash medium moving | 0.92 | 1.10 | 0.15 | -0.89 | 2.93 |
| Flash short | 0.81 | 0.96 | 0.13 | -1.24 | 2.53 |
| Flash short moving | 0.28 | 0.97 | 0.13 | -1.28 | 2.98 |
